# Differences between left- and right-handers in approach/avoidance motivation: influence of consistency of handedness measures

**DOI:** 10.3389/fpsyg.2014.00134

**Published:** 2014-02-20

**Authors:** Scott M. Hardie, Lynn Wright

**Affiliations:** Evolutionary and Biological Approaches to Behaviour Research Group, School of Social and Health Sciences, University of AbertayDundee, UK

**Keywords:** EHI, consistency, BIS/BAS, left-handed, inhibition, handedness strength, handedness direction

## Abstract

Hand preference is often viewed as a troublesome variable in psychological research, with left-handers routinely excluded from studies. Contrary to this, a body of evidence has shown hand preference to be a useful variable when examining human behavior. A recent review argues that the most effective way of using handedness as a variable, is a comparison between individuals who use their dominant hand for virtually all manual activities (consistent handers) versus those who use their other hand for at least one activity (inconsistent handers). The authors contend that researchers should only focus on degree of handedness rather than direction of preference (left versus right). However, we argue that the field suffers from a number of methodological and empirical issues. These include a lack of consensus in choice of cut-off point to divide consistent and inconsistent categories and importantly a paucity of data from left-handers. Consequentially, researchers predominantly compare inconsistent versus consistent right-handers, largely linked to memory, cognition and language. Other research on response style and personality measures shows robust direction of handedness effects. The present study examines both strength and direction of handedness on self-reported behavioral inhibition system (BIS) and behavioral activation system (BAS) scores, using evidence from a large (*N* = 689) dataset including more than 200 left-handers. There were degree of handedness effects on BIS and BAS-Fun Seeking, but effects are largely driven by differences between consistent left-handers and other groups. Choice of cut-off point substantively influenced results, and suggests that unless a suitable sample of left-handers is included, researchers clarify that their degree of handedness effects are applicable only to right-handers. We concur that strength of hand preference is an important variable but caution that differences related to consistency may not be identical in right and left-handers.

## INTRODUCTION

Hand preference has long been viewed as a troublesome variable in much research in psychology; left-handers in particular have been perceived as a noisy and unpredictable population and have often been excluded from studies (e.g., Ferrucci et al., 2013). However, a growing body of evidence suggests that handedness may provide some useful insights into individual differences in behavior (e.g., Wright et al., 2004; Kaploun and Abeare, 2010). Early research tended to examine *differences* between left- and right-handers and often found contradictory influences on behavior, although more recently there has been a move toward examining how handedness categories may influence the relationship with other variables (Beratis et al., 2011; Wright and Hardie, 2012; Hardie and Wright, 2013). In parallel, there is a growing body of research which focuses on strength of handedness, that is, the extent to which individuals favor their chosen hand (regardless of direction of preference). As a consequence, there has been debate in the literature about which of these aspects of handedness researchers should focus upon. The debate has been brought into focus by a recent review. This work contends that the most appropriate way to view handedness is using comparisons between consistent/strong handers who use their chosen hand for virtually all manual activities and inconsistent/mixed handers, who use their other hand for at least one activity (Prichard et al., 2013). Prichard et al. (2013) clearly advocate that “Instead of direction of hand preference being the variable of interest, it should be degree” (p. 1).

While Prichard et al. (2013) provide a very useful synthesis of strength of handedness research and expand our understanding of handedness (especially in the areas of memory retrieval and belief updating/cognitive flexibility); there are problems with the notion that direction is not an appropriate measure. For example, on the basis of an item response theory evaluation of one of the major handedness questionnaires (Oldfield’s, 1971; Edinburgh Handedness Inventory, EHI), Büsch et al. (2010) strongly argue that only two classes of response emerge – left- and right-handed. Other researchers have argued that the most appropriate way to measure handedness is to examine a tripartite model – covering consistent-left, consistent-right and mixed-handers, based on factor-analysis (Peters and Murphy, 1992), neuroimaging (e.g., Knecht et al., 2000; Kirveskari et al., 2006) and behavioral studies (Kaploun and Abeare, 2010). Finally, recent work in this area combines both direction and degree; Lyle et al. (2012a) found differences between consistency groups within handedness categories. This is potentially the most effective way of understanding the influence of handedness on behavior, and may be seen as a “gold standard” for future work, but such studies remain rare.

In order for degree of handedness to be considered as a valid variable, there is a need to examine the empirical and conceptual basis for this measure. After conducting a review of the methodology and theoretical stance of the authors of more than 30 articles using consistency of handedness as a variable, four major issues were identified. The first two are issues which are important for the field to debate and come to a consensus over, and are noted here simply in order to stimulate debate, while the second two issues will be empirically examined.

Firstly, the use of the EHI as a measure of handedness may be criticized. Oldfield’s, (1971) EHI is a self-report measure and respondents answer questions regarding their preference to use a chosen hand “always” “mostly” or to use “either” hand on 10 manual tasks (e.g., writing, throwing). Scores are converted into a Laterality Quotient ranging from -100 (complete left-hand preference) through 0 (no preference) to +100 (complete right-hand preference). This instrument has been extensively evaluated since its’ inception (Bryden, 1977; Williams, 1986; Dragovic, 2004), highlighting problems with the original scoring system (Fazio et al., 2012) which can lead to errors, as well as issues with the structure of the questionnaire itself (Dragovic, 2004). Recently, Milenkovic and Dragovic (2013) proposed that a seven-item version was superior to the original 10-item version, although Veale (2014) disputes this, instead offering her own four-item version. The crux of this debate is that some items may cause considerable measurement error, and that the 10-item version may lead to an overestimation of the proportion of mixed-handers (Dragovic et al., 2008; Büsch et al., 2010). The field needs to address these problems and agree on a standardized way to measure hand preference strength, before an accurate assessment of findings can be made.

Secondly, the use of a split to divide a potentially continuous variable (in this case, strength of handedness) into discrete categories has been criticized for at least the last 30 years (Cohen, 1983; Streiner, 2002; Irwin and McClelland, 2003). MacCallum et al. (2002), for example, argue that it can result in a loss of analytical power or may create falsely significant results. DeCoster et al. (2009) specifically examined the use of dichotomization of samples in psychological research, contacting a number of researchers to establish their rationale for this. The researchers followed this up with Monte Carlo simulations and conclude that continuous variables outperformed dichotomized versions in the majority, but not all of the cases. They produced criteria for dichotomising samples, but it should be noted that the emphasis was on the use of data to support the categorization process. This poses a question for researchers, if dichotomization is being used, should it be done on a seemingly artificial basis (median of hand strength), or should it rely on a split based on underlying latent classes (such as left versus right – Büsch et al., 2010)? Another option might be to use the mean score in each sample and convert the preference scores into stanines, and use stanine-5 exclusion to split the sample. This type of split is used in some areas of psychology (e.g., Moritz et al., 2006) and may be worthy of examination. Alternatively, DeCoster et al. (2009) suggest that extreme group analysis (i.e., selectively recruiting participants from the extremes) is a viable strategy, so perhaps this might be a useful way of testing consistency? The use of a median split for dichotomization needs to be more strongly justified by researchers, perhaps using DeCoster et al. (2011) recommendations.

Assuming that handedness should be examined using a non-continuous categorization, the third issue relates to the choice of the cut-off point to divide populations into consistent versus inconsistent handers (IH). The majority of studies use a notional median value of 80 on the EHI to split their groups into consistent and inconsistent (e.g., Christman and Butler, 2011; Lyle and Orsborn, 2011; Dollfus et al., 2012; Westfall et al., 2012), but it is not clear whether this median value is consistently found within individual samples. It is not common practice in many of these studies to publish their own median values, making the validity of a median split at 80 questionable. Even if the median value is established, there are additional problems with a lack of consistency in how to operationalize the split itself. For example, there are times when the consistency group is defined as scoring above the proposed median, i.e., 85 or above (Propper et al., 2005; Christman et al., 2009; Jasper et al., 2009). There are other times when it is defined as scoring at the median and above, i.e., 80 or above (Christman et al., 2006; Lyle and Grillo, 2014), or occasionally at some other figure such as 95 or above (e.g., Lyle et al., 2008). This lack of consistency across studies makes it difficult to directly compare findings and also suggests that *choice of cut-off* may influence results.

The final, and arguably the most important issue relates to how left-handers fit into this area of research. As a group, left-handers present a challenge to researchers, as they are generally less strongly lateralized than right-handers (Oldfield, 1971) and are relatively scarce, comprising approximately 10% of the general population (Ellis et al., 1988). Even more problematic, is that consistent or strongly lateralized (EHI < –80) left-handers make up only 2–3% of all individuals (Prichard et al., 2013). This makes them an extremely difficult group of participants to recruit, and only a few of the many studies of degree of handedness have been able to recruit a sufficient number of strong left-handers to be able to examine them as a group. As noted by Prichard et al. (2013) this means that the vast majority of this research predominantly compares inconsistent versus consistent groups largely, or exclusively, made up of right-handers (for an exception, see Lyle et al., 2012b). This conflicts with much of the literature which states their findings in terms of consistent versus IH, without making the rightward bias clear in terms of the narrative used in title, introduction and discussion (e.g., Niebauer, 2004; Westfall et al., 2010; Rose and Nagel, 2012). By failing to have enough data on strong left-handers, researchers are not in a sufficiently robust position to be able to say whether they are definitely the same as, or different from, strong right-handers. To clarify this, it is suggested that the field states clearly when the comparison group is predominantly made up of right-handers only (for an example of this approach, see Christman, 2013).

The current study seeks to examine the issues of cut-off point choice and a lack of empirical data from left-handers, in light of Prichard et al.’s (2013) review and their strong assertion that direction of handedness is a “more powerful and appropriate way to classify handedness than the traditional one based on direction (right versus left)” (p. 1). Arguably this assertion is premature, particularly due to a lack of data from consistent left (CL)-handers, and that the studies thus far suffer from a lack of agreement in terms of the cut-off points used to test consistency effects. Consequentially, there is one main research question that requires answering: does strength of handedness influence left- and right-handers in the same way?

In order to do this, the present research examines the influence that strength of handedness has on a dataset which has a relatively large (*N* = 202) number of left-handers, seeking to understand the potential relationship both strength and direction may have on findings. As noted previously, recent work on degree of handedness has been extensively linked to areas of cognition such as memory. The present study extends this into an area of personality, focusing on the relationship between handedness and motivation measured by the behavioral inhibition system (BIS) and behavioral activation system (BAS) scales of Carver and White (1994). The BIS/BAS scales are a self-report measure of the revised reinforcement sensitivity theory of personality (rRST; Gray and McNaughton, 2000). Briefly, this theory postulates that behavior is broadly influenced by three interacting systems; the BAS which motivates approach, reward and impulsivity; the fight-flight-freeze system (FFFS) which relates to fear of a negative outcome, punishment and withdrawal; and the BIS which resolves conflict within or between the other two systems (see Corr and McNaughton, 2008 for details). Prior studies have linked the right-hemisphere to behavioral inhibition and behavioral avoidance (e.g., Davidson, 1985, 1995, 1998), with Sutton and Davidson (1997) describing the left hemisphere as corresponding to approach behavior and the right hemisphere to avoidance behavior. For example, Shackman et al. (2009) have shown that individuals reporting themselves as behaviorally inhibited have an increased resting activity within their right dorsolateral prefrontal cortex. Other work links the right-hemisphere to infants’ temperamental shyness, anxiety, and behavioral inhibition (Schmidt et al., 1999; Fox et al., 2001). Added to this are animal studies linking left-hand preference to delays in exploratory and investigative behavior (Hopkins and Bennett, 1994; Cameron and Rogers, 1999). There is evidence to suggest that measurements of lateral preferences are indicators of hemispheric preferences (Kinsbourne, 1997; Jackson, 2008), with the lateral preference indicative of a preference for the contralateral hemisphere. Previous work in this area (Wright et al., 2009) found hand direction differences, but the evidence has yet to be examined in terms of strength of handedness.

The current study seeks to investigate whether strength of handedness influences left- and right-handers in the same way, in terms of their relationship to BIS/BAS variables. This will be investigated empirically in two ways:

(1) Systematically examining the influence of cut-off points on strength of handedness findings. This will be done by comparing the dataset using a range of cut-off points derived from both the literature and the data itself.
(2) Examining how strength of handedness relates to other variables? This will use regression to examine whether the relationship between variables is the same for both handedness groups.

## MATERIALS AND METHODS

### PARTICIPANTS

Six hundred and eighty-nine participants took part in this study, 272 were male and 417 were female. The majority (*N* = 502) were in the 18–29 year category, comprising 76% of males and 71% of females. Two hundred and two were left-handed, 481 were right-handed, and the remaining six had no overall preference.

### MEASURES

Demographics including gender and age category (18–29, 30–39, 40–49, 50–59, 60–69, and 70 + years) were collected. The EHI (Oldfield, 1971) was used to measure strength and direction of handedness, where participants were asked to indicate which hand they would normally use in each of ten tasks. Choices were Left Always, Left Mostly, Either, Right Mostly, Right Always, and as in previous work (Hardie and Wright, 2013) these were scored as -10, -5, 0, 5, and 10, respectively. Totaling this up yielded a score ranging from -100 (completely left-handed) to +100 (completely right-handed).

Carver and White’s (1994) BIS/BAS scale was used to measure self-reported BIS, BAS and FFFS scores. This instrument has 20 items sub-divided into four categories. Three scales measure BAS – Reward Responsiveness (e.g., “It would excite me to win a contest”), Fun Seeking (e.g., “I often act on the spur of the moment”) and Drive (e.g., “I go out of my way to get things I want”. Originally only a single category measured BIS sensitivity (e.g., “Criticism, or scolding hurts me quite a bit”) but this has subsequently been subdivided into FFFS (questions 2 and 22) and BIS scales (remaining five BIS questions) based on previous work (Corr and McNaughton, 2008; Hardie and Wright, 2013). In all cases, questions were answered as one of four options, ranging from “Very false for me” to “Very true for me” and scored as per Carver and White (1994).

### PROCEDURE

Participants were recruited from both university and the general public through a sustained campaign of emails, website notices, recruitment at public science centers, and at science fairs over the course of around 12 months. We paid particular attention to the recruitment of left-handers, asking for people who considered themselves to be “left-handed” but we also recruited people more generally and tested all individuals who agreed to participate regardless of hand preference. Testing was carried out via a web-based presentation of the questionnaires, in a randomized order, after participants agreed to participate. The research was approved by the school research ethics committee and abided by the British Psychological Society Code of Human Research Ethics.

### STATISTICAL ANALYSIS

Statistical analyses were carried out using SPSS v21. *α* was set at 0.05. The strength of hand preference scores were initially examined in terms of median scores, compared by direction of hand preference and gender. This was followed by an investigation of the influence of categorization system on measures of BIS/BAS, with all participants being assigned into Consistent/Inconsistent categories based on six separate classification systems with different cut-off points on the EHI. As gender has been shown to influence BIS/BAS scores, it was also included as a factor in all analyses. For each classification scheme the following three analyses were carried out:

Consistent handedness versus inconsistent handedness regardless of direction [ANOVA 2 (Gender) × 2 (Consistency)].

Consistent left (CL, consistent right (CR), and inconsistent (IH) handers [ANOVA 2 (Gender) × 3 (Consistency)].

Consistent left (CL), inconsistent left (IL), consistent right (CR), and inconsistent right (IR) handers [ANOVA: 2 (Gender) × 4 (Consistency)].

This was undertaken on BIS, FFFS, BAS-Reward Responsiveness (BAS-RR), BAS-Fun Seeking (BAS-FS), BAS-Drive (BAS-D), as well as a combined BAS score. For the second and third analyses, *post hoc* tests with Bonferroni corrections were calculated where a main effect of consistency was found. Only significant results will be reported.

Handedness was also examined in terms of a regression model, undertaking the following regression analyses:

EHI scores to include directionality and absolute scores to assess general relationship to strength.

Left versus right (as has been used in other studies, such as Hardie and Wright, 2013) where the analyses look at each category separately.

As gender was related to most of these measures, stepwise multiple regressions were used to examine the relationship between handedness and BIS/BAS variables. For each of the analyses, step one was to regress the BIS/BAS measures on gender. In step two, the measures of hand strength were introduced. A significant increase in *R*^2^ when comparing the first to second step would indicate that handedness accounts for variance in BIS/BAS measures over and above those related to gender. If EHI is a significant predictor, then direction of handedness is important, while if absolute is significant then strength is most important. Beta weights provide the basis for examining any relationships. The key data is *R*^2^ change and individual beta weights for the variables of interest.

## RESULTS

**Table 1** shows that the cut-off for the entire sample is 60, for left-handers only, it is –65 and for right-handers only, it is 75.

**Table 1:** Distribution of EHI scores.

	Whole DATA set		Absolute strength		Left-handers only		Right-handers only	
	(*N* = 689)		(*N* = 689)		(*N* = 202)*		(*N* = 481)*	
EHI score median	60		70		-65		75	
EHI score mean (SD)	32.43 (64.1)		67.0 (25.8)		-59.0 (25.5)		71.22 (24.1)	

	**Male**	**Female**	**Male**	**Female**	**Male Left**	**Female Left**	**Male Right**	**Female Right**
	(***N* = 272)**	(***N* = 417)**	(***N* = 272)**	(***N* = 417)**	(***N* = 86)**	(***N* = 116)**	(***N* = 186)**	(***N* = 295)**

EHI score median	60	60	70	70	-65	-65	75	75
EHI score mean (SD)	29.9 (65.5)	34.1 (63.2)	68.1 (23.0)	66.3 (27.4)	-60.5 (24.4)	-57.8 (26.3)	71.6 (21.5)	71.0 (25.6)

*Six individuals who had an EHI score of 0 were removed for the left versus right figures.

### EXAMINATION OF THE INFLUENCE OF CATEGORIZATION SYSTEMS

**Table 2** shows variation within handedness categories depending upon the classification system used. The percentage of the sample categorized as consistent left – ranged from 5.6% of the sample in the most stringent to 21.2% in the loosest classification, and for CR-handers, these ranged from 26.9% (stringent) to 57.8% (loosest).

**Table 2:** Influence of split value used, in terms of number of participants in each category.

Median categorization system	Consistent left-handers	Inconsistent left-handers	Consistent right-handers	Inconsistent right-handers
EHI85 (Consistent ± 85)	38 (5.6%)	164	184 (26.9%)	297
EHI80 (Consistent ± 80)	53 (7.8%)	149	235 (34.4%)	246
EHI75 (Consistent ± 75)	66 (9.7%)	136	261 (38.2%)	220
EHI70* (Consistent ± 70)	87 (12.7%)	115	304 (44.5%)	177
EHI60** (Consistent ± 60)	119 (17.4%)	83	367 (53.7%)	114
EHI40 (Strong ± 45)	145 (21.2%)	57	395 (57.8%)	86

*Based on actual median calculated from absolute strength figures.

**Based on actual median calculated from EHI strength figures.

For all variables (except total BAS) gender was a significant factor. Females were significantly higher on BIS, FFFS and BAS-RR, while males were significantly higher on BAS-D and BAS-FS. All are *F*_(1,685)_ > 4.7, with *p* values = 0.035 or lower. There were no interactions between gender and handedness categories. The remaining analyses, therefore, focus on the influence of categorization. There were no main effects using either the EHI60 or EHI40 classifications.

### TWO CATEGORY SPLITS: CONSISTENT (CH) VERSUS INCONSISTENT (IH) HANDERS

**Table 3** shows that CH had a significantly higher FFFS score only when using the EHI85 cut-off point [*F*_(1,685)_ = 12.17, *p* = 0.001]. Looking at BIS scores, CH had significantly higher values in the EHI85 [*F*_(1,685)_ = 7.73, *p* = 0.006], EHI80 [*F*_(1,685)_ = 4.47, *p* = 0.035], and EHI75 [*F*_(1,685)_ = 6.49, *p* = 0.011] classifications. IH had a significantly higher value of BAS-FS, and like the BIS scores, these were only significant in EHI85 [*F*_(1,685)_ = 6.87, *p* = 0.009], EHI80 [*F*_(1,685)_ = 4.78, *p* = 0.029], and EHI75 [*F*_(1,685)_ = 5.46, *p* = 0.020] classifications. There were no other significant effects.

**Table 3:** The influence of median split point and handedness categorization system on significant consistency of handedness differences found on the BIS/BAS scales.

Scale	Finding*	EHI85 Split	EHI80 Split	EHI75 Split	EHI70 Split
**Two categories**
FFFS	CH > IH	0.001			
BIS	CH > IH	0.006	0.035	0.011	
BAS-FS	IH > CH	0.009	0.029	0.020	
**Three categories**
FFFS	CR > IH	0.002			
BIS	CL > IH	0.001	0.046	0.005	0.014
	CL > CR	0.018			
BAS-FS	IH > CL	0.005	0.011	0.007	
BAS-RR	CL > CR			0.037	0.009
	CL > IH				0.046
Four categories**
FFFS	CR > IL	0.008			
	CR > IR	0.035			
BIS	CL > CR	0.036			
	CL > IL	0.010			
	CL > IR	0.001	ns	0.007	0.038
BAS-FS	IL > CL	0.019	0.028	0.017	
	IR > CL	0.013	0.037	0.031	
BAS-RR	CL > CR				0.019

*CH, consistent handers; IH, inconsistent handers; CL, consistent left-handers; CR, consistent right-handers; IL, inconsistent left-handers; IR, inconsistent right-handers.

**Six individuals who had an EHI score of 0 could not be assigned a direction (left or right) and were removed from the four category analyses.

### THREE CATEGORY SPLITS: CONSISTENT RIGHT (CR), CONSISTENT LEFT (CL) AND INCONSISTENT (IH) HANDERS

There was a significant main effect of category on FFFS scores (EHI85), *F*_(2,683)_ = 6.23, *p* = 0.002. *Post hoc* analyses revealed that only CR was significantly higher than IH (*p* = 0.002). For BIS, there was an influence of category on differences from EHI85 through to EHI70, all *F*_(2,683__)_ > 3.34, *p* values = 0.036 or lower. Further analyses showed that CL was significantly different from CR in only the EHI85 system, but differed from IH in all classifications. For BAS-FS, there were main category effects in EHI85, EHI80 and EHI75 – *F*_(2,683)_ > 4.55, with *p* values = 0.011 or lower. In all cases, only CL was significantly lower than IH. There was an additional main effect of categorization on BAS-RR, for both EHI75 [*F*_(2,683)_ = 3.24, *p* = 0.040] and EHI70 [*F*_(2,683)_ = 4.44, *p* = 0.012]. In both cases CL were significantly higher than IH, and in EHI70 CL were also significantly higher than CR.

### FOUR CATEGORY SPLITS: CONSISTENT RIGHT (CR), CONSISTENT LEFT (CL), INCONSISTENT LEFT (IL) AND INCONSISTENT RIGHT (IR) HANDERS

Comparing fully across hand direction and consistency categories helps to clarify where differences are arising (**Table 3**). Again the FFFS main effect is linked to the EHI85 classification only, *F*_(3,675)_ = 4.27, *p* = 0.005 and *post hoc* analyses revealed CR were significantly higher scoring than both IL and IR. The consistent groups did not significantly differ. For BIS, there were significant main effects of classification system from EHI85 through to EHI70, all with *F*_(3,675)_ > 2.66, *p* values = 0.047 or lower. In all cases CL were significantly greater than IR, except for EHI80 (*p* = 0.052). For EHI85, CL were also significantly higher in BIS than CR and IL. There were also main effects of classification system on BAS-FS scores for EHI85, EHI80 and EHI75 categories [all *F*_(3,675)_ > 3.13, *p* values = 0.025 or lower]. In all three cases, CL were significantly lower than both IL and IR. Finally, for EHI70 there was also a difference in BAS-RR scores, *F*_(3,675)_ = 3.07, *p* = 0.027, where only the CL group had significantly higher scores than CR (*p* = 0.019).

### RELATIONSHIP OF STRENGTH OF HAND PREFERENCE TO OTHER VARIABLES

As the previous analyses predominantly found differences in BIS and BAS-FS then the following analyses are limited to these.

### STRENGTH OF HANDEDNESS

In step 1 (Gender) the model successfully predicted BIS [*F*_(1,688)_ = 50.22, *p* < 0.0001, *R*^2^ = 0.07] and BAS-FS [*F*_(1,688)_ = 8.24, *p* = 0.004, *R*^2^ = 0.01] and was also the case in step 2 (Gender and Handedness), for both BIS [*F*_(3,688)_ = 19.85, *p* < 0.0001, *R*^2^ = 0.08] and BAS-FS [*F*_(3,688__)_ = 4.11, *p* = 0.007, *R*^2^ = 0.02]. In the case of BIS, the introduction of handedness in Step 2 significantly improved the model, *F*_(2,685)_ = 4.41, *p* = 0.012, Δ*R*^2^ = 0.012. Both EHI [β = –0.088, *t*(688) = –2.26, *p* = 0.024] and absolute strength [β = 0.101, *t*(688) = 2.59, *p* = 0.010] were significant but weak predictors of BIS. In the case of BAS-FS, handedness failed to significantly improve the model, and absolute strength was not a significant predictor. In general, handedness only explained a very small amount of overall variance.

### STRENGTH OF HANDEDNESS FOR EACH CATEGORY

The sample was divided into left- and right-handers in order to investigate the relationship between BIS-measures and handedness strength separately. In this case, only absolute strength is used, as for each handedness group this is effectively the same figure.

#### Right-handers (i.e., EHI scores > 0, N = 481)

Neither BIS nor BAS-FS were significantly correlated with absolute strength. The model successfully predicted BIS in both Step 1 [*F*_(1,480)_ = 47.96, *p* < 0.0001, *R*^2^ = 0.09] and Step 2 [*F*_(2,480)_ = 24.15, *p* < 0.0001, *R*^2^ = 0.09] but the introduction of strength of handedness in Step 2 did not significantly improve the model. The model failed to successfully predict BAS-FS.

#### Left-handers (i.e., EHI scores < 0, N = 202)

BIS significantly correlated with absolute hand strength *r*(202) = 0.140, *p* = 0.024, but BAS-FS did not. BIS was successfully predicted in both Step 1 [*F*_(1,201)_ = 7.95, *p* = 0.005, *R*^2^ = 0.04] and Step 2 [*F*_(2,201)_ = 6.44, *p* = 0.002, *R*^2^ = 0.06] and the introduction of strength of handedness in Step 2 significantly improved the model *F*_(1,199)_ = 4.78, *p* = 0.030, Δ*R*^2^ = 0.023, with strength of handedness a significant predictor of BIS [*β* = 0.150, *t*(201) = 2.19, *p* = 0.030]. BAS-FS was also successfully predicted in both Step 1 [*F*_(1,201)_ = 5.32, *p* = 0.022, *R*^2^ = 0.03] and Step 2 [*F*_(2,201)_ = 3.80, *p* = 0.024, *R*^2^ = 0.04] but introduction of handedness in Step 2 did not significantly improve the model.

## DISCUSSION

The current study did not find a median hand preference score of 80, a value which has been used in most previous studies (e.g., Christman et al., 2008; Westfall et al., 2010; Lyle et al., 2012a). The present sample comprises a relatively large sample of left-handers added to a sample of more than 400 right-handers, which yielded a median strength of 60. By taking absolute score (strength regardless of direction) this increases, but even examining only right-handers there is a median of 75. This calls into question the robustness of using a fixed value cut-off point based on a notional median score, and also illustrates the potential confound that may arise when using an actual versus notional median value. Differences in scoring of the EHI may potentially be a factor, as the original scoring system is problematic (Fazio et al., 2012). However, most researchers in the field appear to use a system dividing strength into multiples of 5 ranging from –10 for left-always to +10 for right-always (e.g., Christman et al., 2008; Lyle et al., 2012a; Hardie and Wright, 2013), so it is unlikely that scoring differences greatly influenced the current results.

Examining the influence of strength and direction of handedness on measures of Carver and White’s (1994) BIS/BAS scales demonstrated that consistency of handedness had an influence on several measures. These were mainly related to BIS and BAS-FS which were significantly different across three different split points (EHI85, EHI80, and EHI75), suggesting that these were robust differences. As mentioned above, the current study did not find a median score of 80, so the use of this as a cut-off point may be questioned. When using a notional median of 80, which a majority of studies do (e.g., Jasper et al., 2008; Christman and Butler, 2011; Lyle and Orsborn, 2011; Westfall et al., 2012), the present study demonstrated some strong differences between CH and IH especially when using an “above median” cut-off of 85 (e.g., Propper et al., 2005; Jasper et al., 2009). For BIS scores, using the three category model (CR, CL, and IR) then CL were higher in BIS than the other two groups, suggesting that this group were strongly influencing the findings. In the four category classification (additionally splitting IH into IL and IR), CL were again significantly higher in BIS than the other three categories. Therefore, by selectively choosing this cut-off point to determine consistent handers, the current research findings could be interpreted as strongly arguing that consistent left-handers were significantly higher in BIS than other handedness groups and suggests that the increased behavioral inhibition of left-handers (e.g., Wright et al., 2009) may be driven by this group. This illustrates that when examined in a sufficient number, consistent left- and right-handers may differ from each other, supporting the contention that in the absence of enough data on left-handers other studies should not automatically assume similarity in behavior.

As expected, it was clear that the choice of cut-off point influenced the extent to which consistency effects on BIS/BAS were shown. This is an important finding, as comparing across other handedness consistency research there is a range of cut-off values for defining the “consistent hander” group, mainly equivalent to EHI85, EHI80, and EHI75 classifications used here. The present work is the first demonstration of the direct effect of choice of how to operationalize the median split point within the same dataset, highlighting the influence and also the need for consensus. A wider examination of the use of median splits across psychology yields a similarly mixed picture. A large proportion of them are largely silent in terms of how a median split is operationalized, for example, indicating that the variable in question was split into two groups based on the median, but not stating what was done with those at the median (e.g., Rydstedt et al., 2008; Tops and Bokshem, 2011; McCullough et al., 2012; Smith et al., 2013). It is common practice to have the split at a point scoring above the median (St Clair-Thomson and Sykes, 2010), and only a few studies indicate what is done with those falling at the median, usually adding them to the “low” group (e.g., Whaley, 2003; Hochwälder, 2009). If the field were to follow this convention, and assuming that the median of 80 can be justified, then this would equate to a consistency cut-off point of >80 on the EHI. As most handedness researchers use the EHI in a Likert scale format, this would equate to using the EHI85 system from the present study and it is suggested that this may be an appropriate way to create strength of preference categories.

Examining direction of handedness influences, only a handful of studies have had a sufficiently large sample of consistent left-handers in order to carry out the “gold standard” analysis of comparing all four groups (Lyle et al., 2012a,b; current study). Unfortunately, this means that for the majority of the literature, the position of consistent left-handers is somewhat confused. In some cases they are dropped from analysis as there is “evidence that strong left- and strong right-handers differ from one another…”**(Christman and Butler, 2011, p. 18), or that “strongly left-handed differ from both the strongly right- and the mixed-handed, and thus may constitute their own group” (Propper et al., 2005, p. 754). In other cases they are subsumed because “strong left-handers resemble strong right-handers, with mixed-handers being distinct from the other two strongly handed groups”**(Christman et al., 2009, p. 1184). This ambiguity is clearly demonstrated in Prichard et al.’s (2013, p. 3) comprehensive review, where they argue that researchers should not consider direction, while paradoxically acknowledging that most of the studies in their review “compared ICH with CR-handers.” Arguably, the clearest position to take is to conduct the “gold standard” test if at all possible, but should there not be enough left-handers to test for this, to clearly state that the difference is based on mainly CR-handers.

In terms of the handedness related differences it appears that both strength and direction of handedness may both relate to BIS/BAS. A relationship between left-handedness and behavioral inhibition has become quite well established, through behavioral studies (Wright et al., 2004, 2013; Wright and Hardie, 2011), self-reported measures (Wright et al., 2009; Lyle et al., 2012a; Hardie and Wright, 2013), comparative evidence (Cameron and Rogers, 1999; Rogers, 2009) and models of hemispheric specialization linking the left-hemisphere to avoidance and the right-hemisphere to approach (see Rutherford and Lindell, 2011 for a review). Previous research found that consistent handers showed significantly higher behavioral inhibition than IH, which might be expected (e.g., Niebauer, 2004; Lyle et al., 2012a). When the present study examined the findings in terms of a relationship to direction of handedness as well (using EHI85) it clearly demonstrated that high scoring CL-handers have the highest mean BIS scores; that for left-handers regression analysis showed hand-strength was a significantly positive but weak predictor of BIS, and unsurprisingly BIS and hand strength were significantly correlated. Taken together, these findings suggest that for left-handers their relationship with behavioral inhibition links to degree of handedness in a way that is different from right-handers.

BAS-FS differences were also found, although these were in the opposite direction, with IH scoring higher than consistent. This is not surprising, as there is a body of evidence suggesting that IH are less conservative (Lyle and Grillo, 2014), more gullible (Christman et al., 2008), more open to non-standard ideas (Barnett and Corballis, 2002; Christman, 2013) and generally more risk aversive (Christman et al., 2007). Similar to the BIS findings, the main differences were largely driven by the influence of CL-handers being significantly different from all IH, but in this case, not CR-handers. This meant that regression analysis did not significantly differ between right- and left-handed groups. High BAS-FS has been linked to instant gratification and lack of future contemplation (Heym and Lawrence, 2010) and trait impulsivity (Smillie et al., 2006), and fits with behavioral evidence that left-handers may show an initial response delay when confronted with novelty (Wright et al., 2013).

Putting this together, the current study shows that left-handers may be different in some aspects of personality, compared to right-handers. The extent to which this can be directly applied to other areas of personality is not currently clear, mainly due to the paucity of data on left-handers. Therefore, the present work will hopefully act as a catalyst for other researchers to collect data from a sufficient number of left-handers, so that future hand preference findings are driven by data, rather than assumptions. What can be generalized to other work is our overall finding that consistent left- and right-handers may not always behave in the same way. This is because it contrasts with other work which argues that direction is not important (Prichard et al., 2013), and potentially leaves a question as to how should the field proceed? The recent work of Lyle et al. (2012b) offers some insight here, as although they found consistent left- and right-handers did not differ in terms of memory accuracy, they did find that left-handers as a group were slower to make judgments about memory. In other work Propper et al. (2007) found that consistent left-handers had a different pattern of sleep compared to CR-handers, and Lyle et al. (2012a) found that CR-handers were more anxious than IH, but for left-handers consistency did not relate to anxiety. As anxiety is seen as an outward sign of BIS activity (Corr and McNaughton, 2008) then the finding of Lyle et al. (2012a) resonates with the current results, and they give the intriguing possibility that direction may be important for left-handers and that strength may be important for right-handers.

The current research also has direct implications for the main theory for how hand strength may influence behavior. This theory relies upon the notion of an increased access to the right-hemisphere for IH (compared to consistent handers), allowing them to better coordinate across both hemispheres, that is, having better interhemispheric interaction (Christman et al., 2004; Niebauer, 2004; Propper et al., 2005; Jasper et al., 2008). When taken from the viewpoint of right-handers these arguments are more or less the same, but by adding consistent left-handers to the equation then these become potentially separable issues.

Indeed, for left-handers the right-hemisphere access argument can also be questioned due to anatomical evidence. This suggests that there are structural asymmetries in the central sulcus, where the dorsolateral motor cortex of right-handers is larger in the left-hemisphere, while the opposite is found for left-handers (e.g., Amunts et al., 2000; Klöppel et al., 2010). Additionally, the contralateral motor control arrangement of the primary motor areas of the brain means that left-hand action is largely operationalized via the right-hemisphere (Grabowska et al., 2012), making *increased *right-hemisphere access arguments for mixed-handers untenable for left-handers. Also contrary to the “IH having an increased right-hemisphere access” model is work by Cherbuin and Brinkman (2006). Using Poffenberger’s Paradigm, they found that for left-handers, increases in hand strength were related to increases in efficiency of interhemispheric interaction and as a group, strong left-handers had the highest accuracy (in letter-matching within and across visual fields), while strong right-handers had the lowest. On the other hand, arguments relating degree of handedness to interhemispheric interaction may be important. For example, Potter and Graves (1988) argued that CR-handers had a poorer interhemispheric transfer performance during a line drawing task when compared with non-right-handers. Luders et al. (2010) showed a negative association between corpus callosum size and strength of handedness, regardless of direction of handedness. This largely supports the idea that strength of handedness may demonstrate something important about how the hemispheres interact.

However, taking a wider examination of evidence, then it becomes apparent that compared to right-handers, left-handers appear to be more heterogeneous in terms of hemispheric organization and specialization (see Hervé et al., 2013, for a recent review). The assumption of consistent left-handers being similar to strong right-handers in interhemispheric connectivity is certainly open to debate, for example, Westerhausen et al. (2004) examined the corpus callosum, and found that left-handers had a higher density of fibers, suggesting greater interhemispheric connectivity. Other recent evidence examining motor control in the primary motor cortex, found that left-handers responded differently from right-handers when transcranial magnetic stimulation (TMS) was applied to either the dominant or non-dominant side (van den Berg et al., 2011). Right-handers were more disrupted in the task when the non-dominant side was stimulated, but left-handers split into two distinct groups – one more disrupted by non-dominant side stimulation, the other by dominant side. A similar conclusion has recently been drawn by Lyle et al. (2012a, p. 13) who argue that “consistency-related effects on interhemispheric interaction may not be the same among left-handers as among right-handers.” In their review, Hervé et al. (2013) make suggestions about future research questions, these include; investigating if left-handers as a group have a different neural organization than right-handers; do left-handers show variation in their intrinsic brain connectivity and how can structural and/or functional asymmetries be related to cognitive functioning in left-handers? Taken together, this suggests that the present theoretical underpinning of degree of handedness differences, while applicable to right-handers may need to be further investigated and/or re-evaluated when considering left-handers.

## LIMITATIONS

The current study has some limitations, including the presentation of questionnaires using a web-based approach. By using this medium, there is the potential problem of participants not responding accurately or honestly. However, while this could occur within the dataset, there is no *a priori* reason to expect that the rate of error would differ according to handedness category, so the main findings should be robust. In addition, although web-based data was collected, the initial recruitment of participants was made before passing on the survey link, meaning that there was some degree of control of the process. It is therefore acknowledged that there will be an element of self-selection in terms of willingness to participate, but again there is no strong reason to believe that this would introduce a bias that would artificially create handedness based results. In order to improve accuracy from self-report questionnaires, future work should include either a pre-existing lie scale or add validity questions, to allow for removal of any clearly invalid responses (Fervaha and Remington, 2013). Finally, the regression results suggest that overall; strength of handedness is a very weak predictor of personality, while direction of handedness seems to demonstrate robust differences between left- and right-handers. This suggests the need for a much wider investigation of the validity of strength of handedness as a predictor.

## CONCLUSION

The present study reinforces the view that consistent left- and right-handers do not always behave in the same way. The clear implication is that researchers need to gather sufficient data on consistent left-handers in order to delineate where behavior either converges with, or diverges from, right-handers. It also highlights the need for the handedness research community to be able to robustly defend the dichotomization of hand consistency on the basis of a strong theoretical and empirical evidence base, including an agreed split-point.

## Conflict of Interest Statement

The authors declare that the research was conducted in the absence of any commercial or financial relationships that could be construed as a potential conflict of interest.

## References

[B1] Amunts K. Jancke L. Mohlberg H. Steinmetz H. Zilles K. (2000). Interhemispheric asymmetry of the human motor cortex related to handedness and gender. *Neuropsychologia* 38 304–312 10.1016/S0028-3932(99)00075-5 10678696

[B2] Barnett K. J. Corballis M. C. (2002). Ambidexterity and magical ideation. *Laterality* 7 75–84 10.1080/13576500143000131 15513189

[B3] Beratis I. N. Rabavilas A. D. Papadimitriou G. N. Papageorgiou C. (2011). Eysenck’s model of personality and psychopathological components in right- and left-handers. *Pers. Individ. Differ.* 50 1267–1272 10.1016/j.paid.2010.10.033

[B4] Bryden M. P. (1977). Measuring handedness with questionnaires. *Neuropsychologia* 15 617–624 10.1016/0028-3932(77)90067-7 896019

[B5] Büsch D. Hagemann N. Bender N. (2010). The dimensionality of the Edinburgh handedness inventory: an analysis with models of the item response theory. *Laterality* 15 610–628 10.1080/13576500903081806 19662566

[B6] Cameron R. Rogers J. L. (1999). Hand preference of the common marmoset (*Callithrix jacchus*): problem solving and responses in a novel setting. *J. Comp. Psychol.* 113 149–157 10.1037/0735-7036.113.2.149

[B7] Carver C. S. White T. L. (1994). Behavioral inhibition, behavioral activation, and affective responses to impending reward and punishment: the BIS/BAS scales. *J. Pers. Soc. Psychol.* 67 319–333 10.1037/0022-3514.67.2.319

[B8] Cherbuin N. Brinkman C. (2006). Hemispheric interactions are different in left-handed individuals. *Neuropsychology* 20 700–707 10.1037/0894-4105.20.6.700 17100514

[B9] Christman S. D. (2013). Handedness and “earedness”: strong right-handers are less likely to prefer obscure musical genres. *Psychol. Music* 41 89–96 10.1177/0305735611415751

[B10] Christman S. D. Butler M. (2011). Mixed-handedness advantages in episodic memory obtained under conditions of intentional learning extend to incidental learning. *Brain Cogn.* 77 17–22 10.1016/j.bandc.2011.07.003 21807450

[B11] Christman S. D. Henning B. Geers A. L. Propper R. E. Niebauer C. L. (2008). Mixed-handed persons are more easily persuaded and are more gullible: interhemispheric interaction and belief updating. *Laterality* 13 403–426 10.1080/13576500802079646 18608851

[B12] Christman S. D. Jasper J. D. Sontam V. Cooil B. (2007). Individual differences in risk perception versus risk taking: handedness and interhemispheric interaction. *Brain Cogn.* 63 51–58 10.1016/j.bandc.2006.08.001 16971031

[B13] Christman S. D. Propper R. E. Brown T. J. (2006). Increased interhemispheric interaction is associated with earlier offset of childhood amnesia. *Neuropsychology* 20 336–345 10.1037/0894-4105.20.3.336 16719626

[B14] Christman S. D. Propper R. E. Dion A. (2004). Increased interhemispheric interaction is associated with decreased false memories in a verbal converging semantic associates paradigm. *Brain Cogn.* 56 313–319 10.1016/j.bandc.2004.08.005 15522769

[B15] Christman S. D. Sontam V. Jasper J. D. (2009). Individual differences in ambiguous figure perception: degree of handedness and interhemispheric interaction. *Perception* 38 1183–1198 10.1068/p6131 19817151

[B16] Cohen J. (1983). The cost of dichotimization. *Appl. Psychol. Meas.* 7 426–443 10.1177/014662168300700301

[B17] Corr P. J. McNaughton N. (2008). “Reinforcement sensitivity theory and personality,” in *The Reinforcement Sensitivity Theory of Personality* ed. Corr P. J. (Cambridge: Cambridge University Press) 155–187 10.1017/CBO9780511819384.006

[B18] Davidson R. J. (1985). “Affect, cognition, and hemispheric specialization,” in *Emotions, Cognition, and Behavior* eds Izard C. E. Kagan J. (New York: Cambridge University Press) 320–365

[B19] Davidson R. J. (1995). “Cerebral asymmetry, emotion and affective style,” in *Brain Asymmetry* eds Davidson R. J. Hugdahl K. (Cambridge, MA: MIT Press) 361–387

[B20] Davidson R. J. (1998). Affective style and affective disorders: perspectives from affective neuroscience. *Cogn. Emot.* 12 307–330 10.1080/026999398379628

[B21] DeCoster J. Gallucci M. Iselin A. R. (2011). Best practices for using median splits, artificial categorization, and their continuous alternatives. *J. Exp. Psychopathol.* 2 197–209 10.5127/jep.008310

[B22] DeCoster J. Iselin A. R. Gallucci M. (2009). A conceptual and empirical examination of justifications for dichotomization. *Psychol. Methods* 14 349–366 10.1037/a0016956 19968397

[B23] Dollfus S. Alary A. Razafimandimby A. Prelipceanu D. Rybakowski J. K. Davidson M. (2012). Familial sinistrality and handedness in patients with first episode schizophrenia: the EUFEST study. *Laterality* 17 217–224 10.1080/1357650X.2011.558510 22385143

[B24] Dragovic M. (2004). Towards an improved measure of the Edinburgh Handedness Inventory: a one-factor congeneric measurement model using confirmatory factor analysis. *Laterality* 9 411–419 10.1080/13576500342000248 15513238

[B25] Dragovic M. Milenkovic S. Hammond G. (2008). The distribution of hand preference is discrete: a taxometric examination. *Br. J. Psychol.* 99 445–459 10.1348/000712608X304450 18447970

[B26] Ellis S. J. Ellis P. J. Marshall E. (1988). Hand preference in a normal population. *Cortex* 24 157–163 10.1016/S0010-9452(88)80025-X 3371012

[B27] Fazio R. Coenen C. Denney R. L. (2012). The original instructions for the Edinburgh Handedness Inventory are misunderstood by a majority of participants. *Laterality* 17 70–77 10.1080/1357650X.2010.532801 21557130

[B28] Ferrucci R. Brunoni A. R. Parazzini M. Vergari M. Rossi E. Fumagalli M. (2013). Modulating human procedural learning by cerebellar transcranial direct current stimulation. *Cerebellum* 12 485–492 10.1007/s12311-012-0436-9 23328908

[B29] Fervaha G. Remington G. (2013). Invalid responding in questionnaire-based research: implications for the study of schizotypy. *Psychol. Assess.* 25 1355–1360 10.1037/a0033520 23815113

[B30] Fox N. A. Henderson H. A. Rubin K. H. Calkins S. D. Schmidt L. A. (2001). Continuity and discontinuity of behavioural inhibition and exuberance: psychophysiological and behavioural influences across the first four years of life. *Child Dev.* 72 1–21 10.1111/1467-8624.00262 11280472

[B31] Grabowska A. Gut M. Binder M. Forsberg L. Rymarczyk K. Urbanik A. (2012). Switching handedness: fMRI study of hand motor control in right-handers, left-handers and converted left-handers. *Acta Neurobiol. Exp.* 72 439–451 10.55782/ane-2012-1914 23377273

[B32] Gray J. A. McNaughton N. (2000). *The Neuropsychology of Anxiety: An Enquiry into the Functions of the Septo-hippocampal System*, 2nd Edn. Oxford, UK: Oxford University Press

[B33] Hardie S. M. Wright L. (2013). The relationship between Revised Reinforcement Sensitivity Theory (rRST), handedness and indecision. *Pers. Individ. Differ.* 55 312–316 10.1016/j.paid.2010.07.021

[B34] Hervé P.-Y. Zago L. Petit L. Mazoyer B. Tzourio-Mazoyer N. (2013). Revisiting human hemispheric specialization with neuroimaging. *Trend Cogn. Sci.* 17 69–80 10.1016/j.tics.2012.12.004 23317751

[B35] Heym N. Lawrence C. (2010). The role of Gray’s revised RST in the P-psychopathy continuum: the relationships of Psychoticism with a lack of fear and anxiety, and increased impulsivity. *Pers. Individ. Differ.* 49 874–879 10.1016/j.paid.2010.07.021

[B36] Hochwälder J. (2009). Burnout among Torgersen’s eight personality types. *Soc. Behav. Pers.* 37 467–480 10.2224/sbp.2009.37.4.467

[B37] Hopkins W. D. Bennett A. J. (1994). Handedness and approach avoidance behavior in chimpanzees (Pan). *J. Exp. Psychol. Anim. Behav. Process.* 20 413–418 10.1037/0097-7403.20.4.413 7964523

[B38] Irwin J. R. McClelland G. H. (2003). Negative consequences of dichotomizing continuous predictor variables. *J. Mark. Res.* 40 366–371 10.1509/jmkr.40.3.366.19237

[B39] Jackson C. J. (2008). When avoidance leads to approach: how ear preference interacts with neuroticism to predict disinhibited approach. *Laterality* 13 333–373 10.1080/13576500802063053 18592433

[B40] Jasper J. D. Barry K. Christman S. D. (2008). Individual differences in counterfactual production. *Pers. Individ. Differ.* 45 488–492 10.1016/j.paid.2008.05.026

[B41] Jasper J. D. Prothero M. Christman S. D. (2009). I’m not sexist!!! Cognitive dissonance and the differing cries of mixed- and strong-handers. *Pers. Individ. Differ.* 47 268–272 10.1016/j.paid.2009.03.010

[B42] Kaploun K. A. Abeare C. A. (2010). Degree versus direction: a comparison of four handedness classification schemes through the investigation of lateralised semantic priming. *Laterality* 15 481–500 10.1080/13576500902958871 19536687

[B43] Kinsbourne M. (1997). “Mechanisms and development of hemispecialization in children,” in *Handbook of Clinical Child Neuropsychology* 2nd Edn eds Fletcher-Janzen E. Reynolds C. R. (New York: Plenum) 102–119 10.1007/978-1-4757-5351-6_5

[B44] Kirveskari E. Salmelin R. Hari R. (2006). Neuromagnetic responses to vowels vs. tones reveal hemispheric lateralisation. *Clin. Neurophysiol.* 117 643–648 10.1016/j.clinph.2005.11.001 16403672

[B45] Klöppel S. Mangin J.-F. Vongerichten A. Frackowiak R. S. J. Siebner H. R. (2010). Nurture versus nature: long-term impact of forced right-handedness on structure of pericentral cortex and basal ganglia. *J. Neurosci.* 30 3271–3275 10.1523/JNEUROSCI.4394-09.2010 20203186 PMC6634103

[B46] Knecht S. Dräger B. Deppe M. Bobe L. Lohmann H. Flöel A. (2000). Handedness and hemispheric language dominance in healthy individuals. *Brain* 123 2512–2518 10.1093/brain/123.12.2512 11099452

[B47] Luders E. Cherbuin N. Thompson P. M. Gutman B. Anstey K. J. Sachdey P. (2010). When more is less: associations between corpus callosum size and handedness lateralization. *Neuroimage* 52 43–49 10.1016/j.neuroimage.2010.04.016 20394828 PMC2903194

[B48] Lyle K. B. Chapman L. J. Hatton J. L. (2012a). Is handedness related to anxiety? New answers to an old question. *Laterality* 18 520–535 10.1080/1357650X.2013.783044 23003219

[B49] Lyle K. B. Hanaver-Torrez S. D. Hacklander R. P. Edlin J. M. (2012b). Consistency of handedness, regardless of direction, predicts baseline memory accuracy and potential for memory enhancement. *J. Exp. Psychol. Learn. Mem. Cogn.* 38 187–193 10.1037/a0024831 21843021

[B50] Lyle K. B. Grillo M. C. (2014). Consistent-handed individuals are more authoritarian. *Laterality* 19 146–163 10.1080/1357650X.2013.783044 23586369

[B51] Lyle K. B. Orsborn A. E. (2011). Inconsistent handedness and saccade execution benefit face memory without affecting interhemispheric interaction. *Memory* 19 613–624 10.1080/09658211.2011.595418 21919589

[B52] Lyle K. B. McCabe D. P. Roediger H. L. (2008). Handedness is related to memory via hemispheric interaction: evidence from paired associate recall and source memory tasks. *Neuropsychology* 22 523–530 10.1037/0894-4105.22.4.523 18590363

[B53] MacCallum R. C. Zhang S. Preacher K. J. Rucker D. D. (2002). On the practice of dichotomization of quantitative variables. *Psychol. Methods* 7 19–40 10.1037/1082-989X.7.1.19 11928888

[B54] McCullough M. E. Cartera E. C. DeWall C. N. Corrales C. M. (2012). Religious cognition down-regulates sexually selected, characteristically male behaviors in men, but not in women. *Evol. Hum. Behav.* 33 562–568 10.1016/j.evolhumbehav.2012.02.004

[B55] Milenkovic S. Dragovic M. (2013). Modification of the Edinburgh Handedness Inventory: a replication study. *Laterality* 18 340–348 10.1080/1357650X.2012.683196 22747439

[B56] Moritz S. Jacobsen D. Willenborg B. Jelinek L. Fricke S. (2006). A check on the memory deficit hypothesis of obsessive-compulsive checking. *Eur. Arch. Psychiatry Clin. Neurosci.* 256 82–86 10.1007/s00406-005-0605-7 16041557

[B57] Niebauer C. L. (2004). Handedness and the fringe of consciousness: strong handers ruminate while mixed handers self-reflect. *Conscious. Cogn.* 13 730–745 10.1016/0028-3932(92)90110-8 15522629

[B58] Oldfield R. C. (1971). The assessment and analysis of handedness: the Edinburgh Handedness Inventory. *Neuropsychologia* 9 97–113 10.1016/0028-3932(88)90084-X 5146491

[B59] Peters M. Murphy K. (1992). Cluster analysis reveals at least three, and possibly five distinct handedness groups. *Neuropsychologia* 30 373–380 10.1016/0028-3932(92)90110-8 1603300

[B60] Potter S. Graves R. (1988). Is interhemispheric transfer related to handedness and gender? *Neuropsychologia* 26 319–325 10.1016/0028-3932(88)90084-X 3399047

[B61] Prichard E. Propper R. E. Christman S. D. (2013). Degree of handedness, but not direction, is a systematic predictor of cognitive performance. *Front. Psychol*. 4:9 10.3389/fpsyg.2013.00009 PMC3560368 23386836

[B62] Propper R. E. Christman S. D. Olejarz S. (2007). Home-recorded sleep architecture as a function of handedness II: consistent right-versus consistent left-handers. *J. Nerv. Mental Disord.* 95 689–692 10.1097/NMD.0b013e31811f44b8 17700302

[B63] Propper R. E. Christman S. D. Phaneuf K. A. (2005). A mixed handed advantage in episodic memory: a possible role of interhemispheric interaction. *Mem. Cognit.* 33 751–757 10.3758/BF03195341 16248339

[B64] Rogers L. J. (2009). Hand and paw preferences in relation to the lateralized brain. *Phil. Trans. R. Soc. Biol. Sci.* 364 943–954 10.1098/rstb.2008.0225 PMC2666076 19064357

[B65] Rose J. P. Nagel B. (2012). Relation between comparative risk, absolute risk and worry: the role of handedness strength. *J. Health Psychol.* 18 866–874 10.1177/1359105312456325 22956681

[B66] Rutherford H. Lindell A. (2011). Thriving and surviving: approach and avoidance motivation and lateralization. *Emot. Rev.* 3 333–343 10.1177/1754073911402392

[B67] Rydstedt L. W. Cropley M. Devereux J. J. Michalianou G. (2008). The relationship between long-term job strain and morning and evening saliva cortisol secretion among white-collar workers. *J. Occup. Health Psychol.* 13 105–113 10.1037/1076-8998.13.2.105 18393580

[B68] Schmidt L. A. Fox N. A. Schulkin J. Gold P. W. (1999). Behavioral and psychophysiological correlates of self-presentation in temperamentally shy children. *Dev. Psychobiol.* 35 119–135 10.1002/(SICI)1098-2302(199909)35:2<119::AID-DEV5>3.0.CO;2-G 10461126

[B69] Shackman A. J. McMenamin B. W. Maxwell J. S. Greischar L. L. Davidson R. J. (2009). Right dorsolateral prefrontal cortical activity and behavioral inhibition. *Psychol. Sci.* 20 1500–1506 10.1111/j.1467-9280.2009.02476.x 19906125 PMC2858783

[B70] Smillie L. D. Jackson C. J. Dalgleish L. I. (2006). Conceptual distinctions among Carver and White’s (1994) BAS scales: a reward-reactivity versus trait impulsivity perspective. *Pers. Individ. Differ.* 40 1039 10.1016/j.paid.2005.10.012

[B71] Smith D. S. Jones B. C. Allan K. (2013). Socio-sexuality and episodic memory function in women: further evidence of an adaptive “mating mode”. *Mem. Cognit.* 41 850–861 10.3758/s13421-013-0301-1 23389699

[B72] St Clair-Thomson H. Sykes S. (2010). Scoring methods and the predictive ability of working memory tasks. *Behav. Res. Methods* 42 969–975 10.3758/BRM.42.4.969 21139163

[B73] Streiner D. L. (2002). Breaking up is hard to do: the heartbreak of dichotomizing continuous data. *Can. J. Psychiatry* 47 262–266 11987478 10.1177/070674370204700307

[B74] Sutton S. K. Davidson R. J. (1997). Prefrontal brain asymmetry: a biological substrate of the behavioral approach and inhibition systems. *Psychol. Sci.* 8 204–210 10.1111/j.1467-9280.1997.tb00413.x

[B75] Tops M Bokshem M. A. S. (2011). Cortisol involvement in mechanisms of behavioral inhibition. *Psychophysiology* 48 723–732 10.1111/j.1469-8986.2010.01131.x 20874754

[B76] van den Berg F. E. Swinnen S. P. Wenderoth N. (2011). Involvement of the primary motor cortex in controlling movements executed with the ipsilateral hand differs between left and right-handers. *J. Cogn. Neurosci.* 23 3456–3469 10.1162/jocn_a_00018 21452954

[B77] Veale J. (2014). Edinburgh Handedness Inventory – Short Form: a revised version based on confirmatory factor analysis. *Laterality* 19 164–177 10.1080/1357650X.2013.783045 23659650

[B78] Westerhausen R. Kreuder F. Dos Santos S. S. Walter C. Woerner W. Wittling R. A. (2004). Effects of handedness and gender on macro- and microstructure of the corpus callosum and its sub regions: a combined high resolution and diffusion-tensor MRI study. *Cogn. Brain Res.* 21 418–426 10.1016/j.cogbrainres.2004.07.002 15511657

[B79] Westfall J. Jasper J. D. Christman S. D. (2012). Inaction inertia, the sunk cost effect, and handedness: avoiding the losses of past decisions. *Brain Cogn.* 80 192–200 10.1016/j.bandc.2012.06.003 22898591

[B80] Westfall J. Jasper J. D. Zelmanova Y. (2010). Differences in time perception as a function of strength of handedness. *Pers. Individ. Differ.* 49 629–633 10.1016/j.paid.2010.05.036

[B81] Whaley A. L. (2003). Confluent paranoia in African American psychiatric patients: an empirical study of Ridley’s typology. *J. Abnorm. Psychol.* 111 568–577 10.1037/0021-843X.111.4.568 12428770

[B82] Williams S. M. (1986). Factor analysis of the Edinburgh Handedness Inventory. *Cortex* 22 325–326 10.1016/S0010-9452(86)80058-2 3731804

[B83] Wright L. Hardie S. M. (2011). “Not ready to sort it yet”. Revised Reinforcement Sensitivity Theory (rRST) predicts left-handed behavioural inhibition during a manual sorting task.* Laterality* 16 753–767 10.1080/1357650X.2010.521752 22304339

[B84] Wright L. Hardie S. M. (2012). Are left handers really more anxious? *Laterality* 17 629–642 10.1080/1357650X.2011.615126 22973815

[B85] Wright L. Hardie S. M. Rodway P. (2004). Pause before you respond: handedness influences response style on the Tower of Hanoi task. *Laterality* 9 133–147 10.1080/13576500244000265 15382714

[B86] Wright L. Hardie S. M. Wilson K. (2009). Handedness and behavioural inhibition: left-handed females show most inhibition as measured by BIS/BAS self-report. *Pers. Individ. Differ.* 46 20–24 10.1016/j.paid.2008.08.019

[B87] Wright L. Watt S. Hardie S. M. (2013). Influences of lateral preference and personality on behavior toward a manual sorting task. *Pers. Individ. Differ.* 54 903–907 10.1016/j.paid.2013.01.005

